# Dysfunction and toxicity of damaged proteins in the etiology of aging and age-related degenerative and malignant diseases

**DOI:** 10.3325/cmj.2020.61.159

**Published:** 2020-04

**Authors:** Miroslav Radman

**Affiliations:** 1 Mediterranean Institute for Life Sciences (MedILS), Split, Croatia; 2 Naos Institute for Life Sciences, Aix-en-Provence, France; 3 Inserm U-1001, Université Paris-Descartes, Faculté de Médecine Paris-Descartes, Paris, France; 4 Center of Excellence for Science and Technology-Integration of Mediterranean Region (STIM), University of Split, Split, Croatia

## Abstract

Health can be defined as a harmony, or homeostasis, of the activities of thousands of different proteins, whereas aging and diseases result from their disharmony manifested at the levels of cells and tissues. Such disharmony is caused primarily by dysfunction and toxicity of misfolded proteins damaged by oxidation. This is an overview of key data that inspired new concepts allowing interpretation and integration of the scientific literature on aging and age-related diseases. These concepts suggest strategies for prevention and attenuation of age-related degenerative and malignant diseases mimicking the life of super-centenarians.

## Introduction: setting the stage

French Nobel prize physicist Jean Perrin is quoted saying that if medical doctors were given the mission to find out how to look inside live bodies, we still would not have x-rays. This implies that too much direction in the research into the unknown is counterproductive. In the face of current favoritism for applied mission-oriented research stands L. Pasteur’s quote: “there is no applied science – only the application of science.”

The noble mission of biomedical research is the mitigation of diseases. The last half century of biomedical research is the largest investment in science of all times, of both human and financial resources. Yet, the results within its mission are dismal: not a single major age-related disease is curable. A scientist with this track record would never get a new grant. The trend over the decades is that the cost of medicine and biomedical research keep rising exponentially (technologies oblige!), while the incidence of incurable deadly diseases does not decline. Since the subject is human suffering and death, it is matter of ethics to challenge biomedical establishment and question whether the chosen mainstream research approach is adequate for the task.

Here is an attempt to do so by offering an alternative view on the etiology of aging and age-related diseases and proposing a simple approach to mitigate simultaneously nearly all incurable diseases by treating their common root cause and not their downstream consequences (which is the current trend).

In the context of said criticism, shared by many colleagues, I zoom out to common sense and argue that the key problem is – at the outset – the deficit of productive concepts and even the propagation of unproductive concepts. For instance, it is common sense that knowing the biology of a single healthy centenarian would be more useful to global public health than studying a million of sick people. From the biology of a healthy centenarian, we would learn how all of few hundred degenerative and malignant diseases could be avoided or healed, while the study of sick people teaches only about the details of biological damage done by diverse diseases. From studying patients, we learn little to nothing about the cause of disease, only about its consequences.

This introduction summarizes the motivation to write this atypical review, which will put forward new concepts that can integrate and interpret the complexity of the existing literature on aging and age-related diseases. There is a wealth of comprehensive reviews on the subject (eg 1) as well as more advanced description of these concepts (2,3).

## Is there a common root cause of aging and age-related diseases?

Similar rate of age-related increase in the incidence of all age-related diseases and death (about 5th power of time lived) suggests their common biological clock. The existence of healthy centenarians hints even to a likelihood of a common root cause of aging and all age-related diseases (otherwise, the accumulation within one genome of the ensemble of genetic resistances to over one hundred diseases would be highly improbable).

Moreover, if a common cause of aging and age-related diseases exists, as proposed here, then by preventing or reducing such common cause, it should be possible to mitigate all age-related diseases and slow-down the rate of aging. It would be simpler to mitigate all age-related diseases simultaneously than a single one. In such a case, most people could live the life of fellow supercentenarians – up to 120 years – without changing the basic human biology. Only when we acquire the relevant knowledge, extending the lifespan beyond 120 years becomes possible – by changing human biology!

What could be the common cause of diseases responsible for 90% of deaths in economically developed countries? Elsewhere (2-4) and below, I argue that it is the irreversible oxidative damage to the proteome that increases with age and correlates similarly with the fraction of life span of a nematode (max. 3 weeks) to humans (max. 120 years) (5,6). The identification of inborn predisposition to age-related diseases is also proposed (below).

## Latency in phenotypic expression, or onset, of age-related diseases

The difficulty in curing age-related diseases is in part due to the stability of somatically acquired genetic (DNA mutations) and epigenetic (DNA and histone modifications) alterations that are their cause. Hence the current interest in gene modification technologies that can correct mutations. But, in view of always limited precision of such procedures in the face of a huge number and uncertain geography of aberrant cells in tissues and of the wrong nucleotides in the genome, the success of such technologies remains uncertain.

In contrast, the manipulation of the phenotype of such genome alterations (both genetic and epigenetic) appears more promising. Long latency in the onset of disease is common place: (i) mutations predisposing to diseases remain silent for decades (those that remain silent forever are called silent mutations or polymorphisms) and (ii) it took 20-25 years for the onset of carcinomas initiated by atomic bomb explosions in Hiroshima and Nagasaki.

This is to say that for health, we are concerned by phenotype (disease), not genotype – as long as it remains phenotypically silent! Many amino acid substitution mutations in proteins/genes, called polymorphisms, are maintained in an inconsequential (phenotypically silent) state by the activity of chaperone proteins that keep correcting the misfolding induced by mutation (7). This way, a genetically predisposed disease is kept in latency.

Another mechanism for suppressing the onset of disease at tissue level, called cellular parabiosis, has been proposed recently (3). Cellular parabiosis is a trans-cellular complementation of cellular deficiencies and defects by the molecular traffic between neighboring cells – a sort of “cellular solidarity” or tissue homeostasis. Its result is the redistribution of goods and averaging of cell activities within a tissue (see cartoons in ref 3).

Cellular parabiosis is a powerful mechanism that can attenuate functional defects of recessive mutations such as hypoxanthine phosphoribosyl transferase deficiency (via gap junctions) (3,8) and mitochondrial respiration defect (by mitochondrial transfer from neighboring healthy cells via tunneling nanotubes or TnT) (9,10).

There are many structures that can mediate cellular parabiosis: TnT (for transfer of proteins, RNA, mitochondria, Golgi apparatus, lysosomes, but also prions and viruses), gap junctions (for the transfer of ions and metabolites), desmosomes, exosomes, and other extracellular cargo vesicles (3).

## Inflammation and the onset of diseases

A particularly interesting aspect of cellular parabiosis is the role of chronic inflammation in the prevention of cellular parabiosis and therefore the acceleration of the onset of diseases. So far, there is no known age-related disease that is not revealed or stimulated by chronic inflammation (uncitable interviews with over 20 experts). Inflammation is a curious phenomenon because, in clinical practice, it appears so often as a life threatening nonsense. The onset of cancers is notoriously linked with inflammation. Yet, it is highly unlikely that a complex tissue-level process like inflammation would evolve to do harm and kill people. Inhibiting inflammation by immunosuppressants puts the patient at high risk of dying of an infectious disease – many non-lethal infectious diseases become lethal by anti-inflammatory immunosuppressants. Inflammation is clearly a battlefield between bacteria or viruses and the immune system, and there is always collateral damage on a battlefield. In coronavirus infection of the lung epithelium, inflammation does the potentially lethal damage to the lung, called fibrosis. Lung fibrosis consists of dead cell cadavers and piles of aggregated oxidation-damaged extracellular proteins, such as collagen, keratin, and others. Fibrotic lung epithelium cannot exchange O_2_ and CO_2_, hence leading to respiratory deficiency and even death.

Clinicians have noticed that persons surviving generalized systemic inflammation, called sepsis, often get back to the same site, the same disease, they had decades ago and got “cured.” It appears that the disease was “silenced”– set in latency – or healed, rather than cured since a lasting inflammation brought it back (Professor David Grainger, Cambridge University, personal communication).

**Figure 1:**
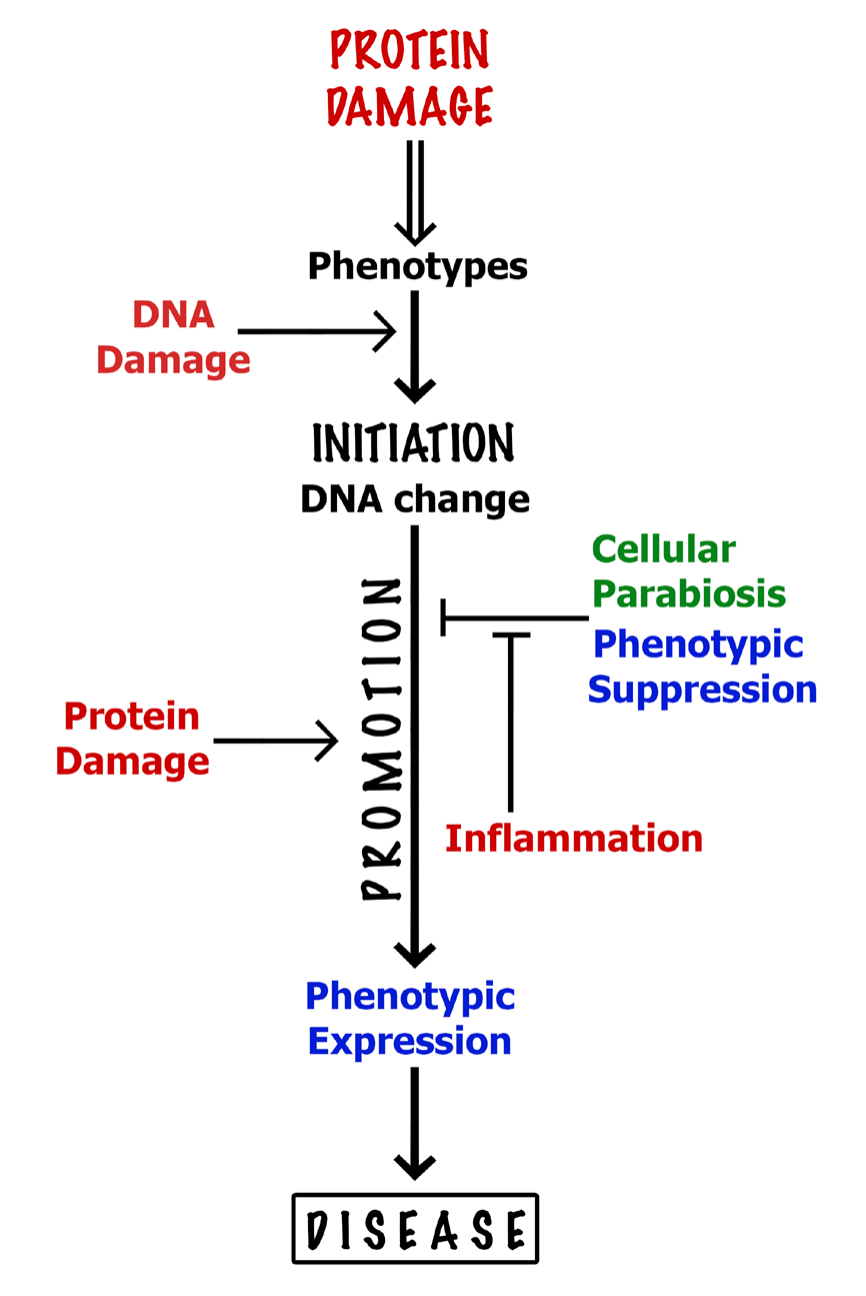
Protein oxidation and misfolding cause age-related diseases. The misfolded or mutant protein is more sensitive to the attack of reactive oxygen species (ROS) and will be oxidised. Two competing processes will come into play, either the misfolding is reversible by the intervention of chaperones and the protein will be folded to its native conformation and keep its function (green arrows); or the oxidative modification (carbonylation) will fix the misfolded conformation by precluding the refolding by chaperones (red arrows). From there, the oxidised and misfolded proteins can follow different paths: 1) the protein is immediately degraded by proteases; 2) the protein avoids degradation and remains in its dysfunctional monomeric state that leads to disease (cancer for example); 3) the protein can form multimeric structures (oligomers and aggregates) that are toxic to the cell and will eventually lead to neurodegenerative diseases, among others. The presence of aggregates causes an immunogenic inflammatory reaction that triggers the onset of age-related diseases.

Sterile inflammations (those not associated with infection) are long-lasting (Figure 1), releasing matrix metalloproteinases and some other serine proteases (eg, plasmin and plasminogen activator) in the extracellular matrix and degrading its constituents, including cell-cell communication channels. Even in cell culture, in the absence of immunity, the addition of nanomolar concentration of powerful inflammation-inducing agent phorbol ester (TPA or PMA) prevents cellular parabiosis (3,8). TPA is the best known tumor promoter, a potent activator of latent cancers initiated by a chemical carcinogen on mouse skin (3). Disease promotion by chronic inflammation is readily explained by the prevention of cellular parabiosis which, itself, appears as the key mechanism of phenotypic silencing of disease, ie, its latency, at the tissue level.

## The root cause of aging and associated diseases is at the level of proteins

With the exception of cancer – which is initiated at single cell level – the frequency of acquired somatic mutations is insufficient to explain the aging of tissues and entire organism. Sequencing of centenarians’ DNA revealed that there are too few mutations to erode proteome activities in cells of aging tissues. However, oxidative damage to proteins, which accumulates exponentially with age in animals and humans (2,5,6,11), is massive enough to ruin the homeostasis of the proteome and cause degenerative and malignant diseases (2).

Degenerative age-related diseases are numerous: neurodegenerative (eg, Alzheimer’s, Parkinson’s, amyotrophic lateral sclerosis – ALS, and several others), metabolic (eg, type 2 diabetes), cardiovascular diseases and conditions, and immunity-related diseases (eg, autoimmune and infectious diseases linked with the weakening of the immune system), all accounting for about one hundred particular diseases and conditions.

Malignant age-related diseases are mostly the solid tumors of epithelia (carcinomas) and some leukemias, totaling also over one hundred of particular diseases.

**Figure 2:**
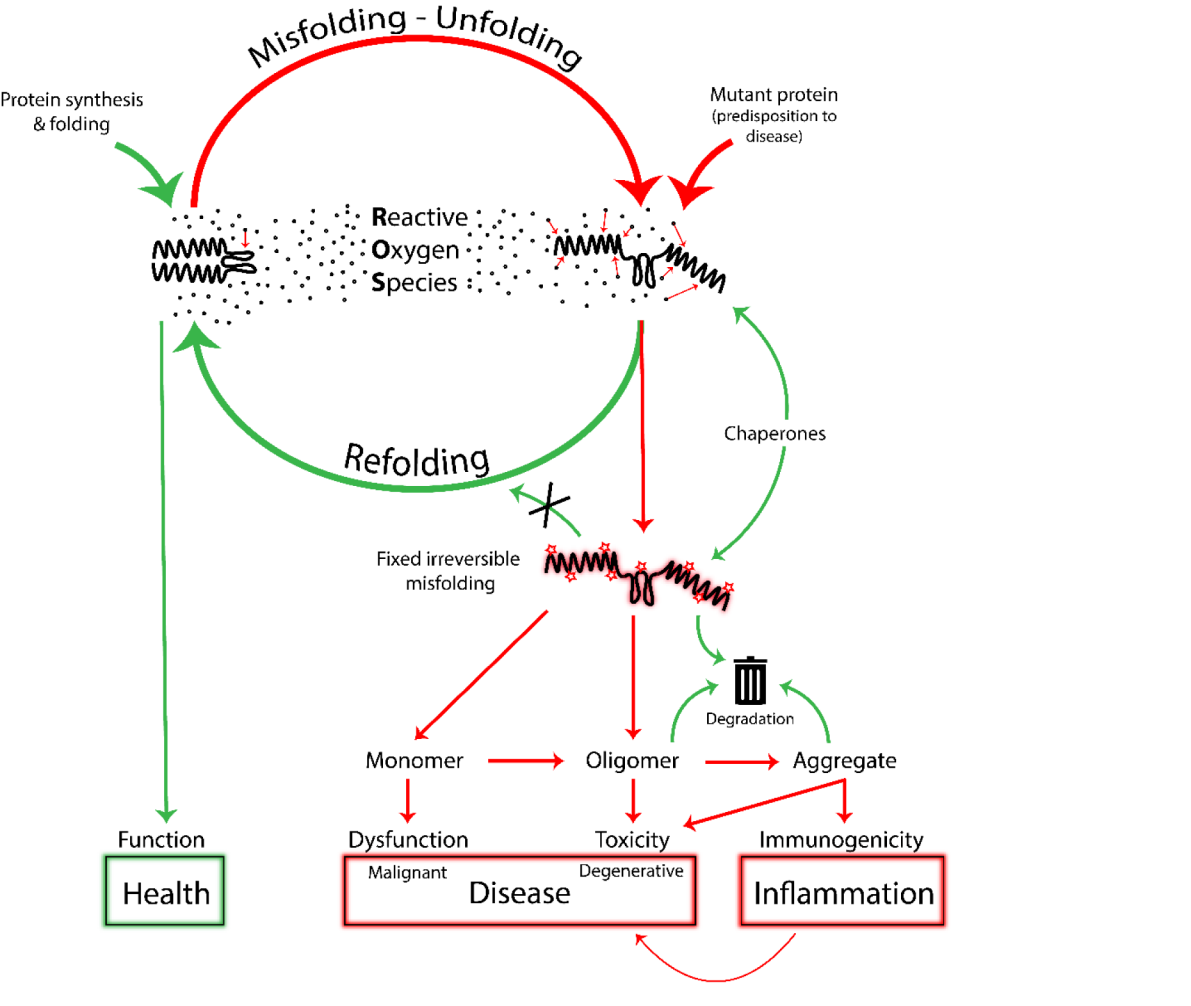
Key molecular and cellular processes affecting the onset of aging and age-related diseases (eg, carcinomas). “Initiation” and “Promotion” are two key mechanistic phases in the onset of age-related diseases. Oxidative proteome damage by reactive oxygen species (ROS) displays diverse reversible phenotypic effects, including generation of stable genetic and epigenetic alterations (“Initiation” by DNA change) kept in phenotypic silence by cellular parabiosis (phenotypic suppression). “Promotion” is typically a chronic inflammation that interrupts cellular parabiosis (see text) leading to phenotypic expression, ie, the onset of disease. DNA damage merely synergises generation of (epi)mutations since protein damage alone is more mutagenic than DNA damage (2,4). Lasting promotion via chronic inflammation activates initiated morbid phenotypes, ranging from malignant growth to cell death. Red colored processes produce, or are affected by, ROS and stimulate initiation and/or promotion of pathological phenotypes, whereas green and blue belong to tissue homeostasis. High adenosine triphosphate and low ROS levels increase proteome quality and promote health and longevity (see text). Figure reproduced from (3).

Therefore, gene-based resistance to each of degenerative and malignant diseases in healthy centenarians would be highly unlikely. But, a protein-based and tissue-based resistance to diseases would include phenotypic silencing of inherited and somatically acquired mutations, and also of the presumably more frequent epigenetic alterations (1,2) (Figure 1 and 2). The concept of tunable and reversible generation of new phenotypes – solely at the level of proteome (oxidative) damage – was demonstrated in bacteria (4). For instance, even DNA mutation rate is a phenotype (mutator or anti-mutator) such that doubling of protein oxidation, at constant reactive oxygen species (ROS), increases the spontaneous mutation rate in *E. coli* by hundred times (4). While DNA damage increases mutation frequency linearly, protein damage does so with the 7th power. Via enzymatic DNA repair, protein damage determines the final level of DNA damage and also the level of replication errors (via mismatch repair). Hence, one could have expected such strong effect of damage to proteins on DNA mutation rates.

## The basic chemistry of aging and death is protein oxidation

This statement relies on the studies of most robust bacteria (eg, *Deinococcus radiodurans*) and animals (eg, Bdelloid rotifers), extremely resistant to radiation and desiccation, which are reviewed elsewhere (2,11-14). A look at Figure 1 in (11) will convince that there is no space for a mechanism of cell death by radiation that does not involve irreversible oxidative protein damage, such as carbonylation. This applies to bacteria and animals.

## From proteins to diseases

In well controlled bacterial model systems, it was shown that the loss of function, or a gain of malfunction, can arise as the result of the overlap of protein misfolding and irreversible oxidation (carbonylation) (4). Proteins of aerobic organisms have evolved structures bestowing resistance to oxidative modification, but even a slight misfolding (due to synthetic error or a silent mutation) is sufficient to lose that resistance. Thus, our proteins are largely “inox” but not all of them: even subtle misfolding will sensitize the protein to oxidation – more than an increase in ROS (in the physiological range) (4).

Carbonylation of a protein will fix (paralyze) its misfolded state that alters its function and generates a phenotypic change coherent with the function of the protein.

Figure 1 shows a key concept: a misfolded protein is subject to two competitive and antagonistic processes – refolding by chaperones into the native oxidation-resistant state and oxidation that will preclude the refolding. Only the oxidized, permanently misfolded proteins matter for pathological phenotypes. The overlap of misfolding and oxidation is crucial for cell pathology and death (2). There are at least three species of damaged proteins involved in pathogenicity:

- The accumulation of oxidized protein “monomers” (ie, the native form) will cause phenotypic change related to its original function. For instance, if related to the maintenance of DNA sequence (DNA replication and repair), malfunction will lead to mutational errors initiating cancer, the so-called driver mutations.

- Misfolded oxidized proteins expose their hydrophobic amino acids, which chase away water molecules between two nearby proteins of the same kind. This will initiate the process of aggregation (Figure 1). Early-stage aggregates are small and are called the oligomers. The smallest oligomers are trimer, the larger ones are circular doughnut-shaped, pore-like, structures. The oligomers of oxidized proteins are hydrophobic (lipophilic) highly cytotoxic structures. They form, or insert in, the biomembranes – both intracellular membranes (eg, of mitochondria, ER, and Golgi) and cellular plasma membrane – causing their depolarization, leakage, and release of inflammatory signals (15). This intracellular process is equivalent to the “home made” production of tools for cell suicide, and massive cell death becomes the cause of degenerative disease (neurodegeneration, sarcopenia, etc). Well characterized toxic oligomers are those of α-synuclein (Parkinson’s disease), SOD1 (ALS), and serum amyloid protein (SAP) (15).

- When oligomers fuse or become linear, they tend to form large to very large aggregates (amyloids, fibrils, etc) that are the well-known hallmarks of neurodegenerative diseases. Aggregates are mostly extracellular but are also seen within the cells (eg, α-synuclein aggregates in motor neurons in Parkinson’s disease, SOD1 aggregates in ALS disease, and amylin aggregates in pancreatic beta-cells in type 2 diabetes).

By themselves, large aggregates are less cytotoxic than suspected earlier; certainly less cytotoxic than the oligomers. But, they may confuse the immune system that can react to them as foreign “non-self” entities and cause auto-immune conditions and diseases (eg, rheumatoid arthritis, multiple sclerosis, Crohn disease, psoriasis, or even type 1 diabetes). Much remains to be done in this area, but the overall picture in Figure 2 is not exceedingly complicated while showing the suspected key events from protein molecules (their folding and intrinsic oxidability) to disease phenotypes (cell loss in degenerative diseases and cell excess in malignant diseases).

While the input toward protein oligomerization and aggregation are the misfolded oxidized proteins, the output are the clean-up systems: proteasomal degradation of monomers and autophagosome-mediated elimination of large aggregates in lysosomes (Figure 1). Oligomers are least prone to either of two sanitation systems, plus they hide in membranes – hence, their persistence and a vicious toxicity. Given the sequence of events drawn in Figure 1, it is evident that proteasome and autophagosome activities determine how much of accumulated precursors for oligomerization and aggregation are available in the cell.

To achieve resilience, best would be to be endowed with high-fidelity protein synthesis, high chaperone activity, low or optimal ROS levels (high ATP/ROS ratio), and highly active proteasome and autophagy!

## Syndromes vs predispositions

We have identified the nature of inborn predispositions to an age-related disease (Parkinson’s disease) as silent mutations in α-synuclein gene (and protein) that increase the susceptibility of α-synuclein to oxidation (2) in proportion to the severity of disease. This was confirmed with five mutant proteoforms of SOD1 predisposing to ALS (unpublished work of Guillaume F. Combes and Marion Giraud). In the nutshell, a phenotypically silent mutation, or polymorphism, that predisposes a protein to oxidation, predisposes the person to a specific age-related disease.

The gist about inborn predisposition is that an initially silent amino acid substitution mutation becomes progressively phenotypic with time, ie, with oxidation, meaning that protein function is destroyed by its oxidation. In contrast, a germ line null (knockout) mutation directly and immediately destroys protein function, the baby is born sick and the disease is a syndrome. Predispositions and syndromes differ by the nature of the mutation: the former mutation is phenotypically latent for decades, the latter is expressed immediately. Therefore, in Figure 1 there is an arrow from “mutant protein” directly to the misfolded protein subject to oxidative damage. Unlike the stochastic errors in protein biosynthesis, a mutation is the same error affecting all molecules of that protein.

Figure 2 places the proposed concepts into general etiology of age-related diseases (see the legend). Figures 1 and 2 show bird-eye view on age-related diseases where entire areas of research (eg, oxidative metabolism, mitochondria, ribosomal fidelity, UPR, telomerase, sirtuins, apoptosis, diverse signaling pathways, etc) are the left-out details of the global picture.

## A daydream: new medicine using prevention and attenuation of age-related diseases

The two key concepts – protein damage and cellular parabiosis (2,3) – open an exciting possibility for mitigation (prevention and attenuation) of all age-related diseases by reducing their most upstream cause: protein misfolding and oxidation. A daydream equivalent to what vaccination and antibiotics did together in mitigating infectious diseases. Curiously, infectious and non-infectious diseases do not appear that separated. Since the immune system weakens and degenerates in its precision and activity, then not only auto-immune diseases, but standard infectious bacterial (eg, pneumonia) and viral (eg, COVID-19) diseases appear also as age-related diseases that increase exponentially with age. They likely share the common cause (2) and mechanism of phenotypic expression (3) of all age-related diseases.

Drug candidates for such mitigation of diseases are the small-molecule hydrophobic antioxidant proteome-shields, inspired by studies by Krisko and Radman (13) and Daly et al (16) and unpublished observations by François-Xavier Pellay in our institute.

A success of this kind would be a significant intervention into our inborn health-related destiny by making us all live long disease-free life – and even die without long painful diseases – which is the mimic of our fellow super-centenarians, who naturally resist all of today’s incurable diseases. What a marvelous project whose success could make thousands of drugs obsolete, in favor of a single class of drugs useful to everybody (to the healthy for prevention, to the sick for healing). A real daydream!
